# Evaluation of multiple micronutrient supplementation and medium-quantity lipid-based nutrient supplementation in pregnancy on child development in rural Niger: A secondary analysis of a cluster randomized controlled trial

**DOI:** 10.1371/journal.pmed.1003984

**Published:** 2022-05-02

**Authors:** Christopher R. Sudfeld, Lilia Bliznashka, Aichatou Salifou, Ousmane Guindo, Issaka Soumana, Irène Adehossi, Céline Langendorf, Rebecca F. Grais, Sheila Isanaka

**Affiliations:** 1 Department of Global Health and Population, Harvard T.H. Chan School of Public Health, Boston, Massachusetts, United States of America; 2 Department of Nutrition, Harvard T.H. Chan School of Public Health, Boston, Massachusetts, United States of America; 3 Epicentre, Niamey, Niger; 4 National Pediatric Hospital of Niamey, Niamey, Niger; 5 Department of Research, Epicentre, Paris, France; Makerere University Medical School, UGANDA

## Abstract

**Background:**

It is estimated that over 250 million children under 5 years of age in low- and middle-income countries (LMICs) do not reach their full developmental potential. Poor maternal diet, anemia, and micronutrient deficiencies during pregnancy are associated with suboptimal neurodevelopmental outcomes in children. However, the effect of prenatal macronutrient and micronutrient supplementation on child development in LMIC settings remains unclear due to limited evidence from randomized trials.

**Methods and findings:**

We conducted a 3-arm cluster-randomized trial (*n* = 53 clusters) that evaluated the efficacy of (1) prenatal multiple micronutrient supplementation (MMS; *n* = 18 clusters) and (2) lipid-based nutrient supplementation (LNS; *n* = 18 clusters) as compared to (3) routine iron–folic acid (IFA) supplementation (*n* = 17 clusters) among pregnant women in the rural district of Madarounfa, Niger, from March 2015 to August 2019 (ClinicalTrials.gov identifier NCT02145000). Children were followed until 2 years of age, and the Bayley Scales of Infant and Toddler Development III (BSID-III) were administered to children every 3 months from 6 to 24 months of age. Maternal report of WHO gross motor milestone achievement was assessed monthly from 3 to 24 months of age. An intention-to-treat analysis was followed. Child BSID-III data were available for 559, 492, and 581 singleton children in the MMS, LNS, and IFA groups, respectively. Child WHO motor milestone data were available for 691, 781, and 753 singleton children in the MMS, LNS, and IFA groups, respectively. Prenatal MMS had no effect on child BSID-III cognitive (standardized mean difference [SMD]: 0.21; 95% CI: −0.20, 0.62; *p* = 0.32), language (SMD: 0.16; 95% CI: −0.30, 0.61; *p* = 0.50) or motor scores (SMD: 0.18; 95% CI: −0.39, 0.74; *p* = 0.54) or on time to achievement of the WHO gross motor milestones as compared to IFA. Prenatal LNS had no effect on child BSID-III cognitive (SMD: 0.17; 95% CI: −0.15, 0.49; *p* = 0.29), language (SMD: 0.11; 95% CI: −0.22, 0.44; *p* = 0.53) or motor scores (SMD: −0.04; 95% CI: −0.46, 0.37; *p* = 0.85) at the 24-month endline visit as compared to IFA. However, the trajectory of BSID-III cognitive scores during the first 2 years of life differed between the groups with children in the LNS group having higher cognitive scores at 18 and 21 months (approximately 0.35 SD) as compared to the IFA group (*p*-value for difference in trajectory <0.001). Children whose mothers received LNS also had earlier achievement of sitting alone (hazard ratio [HR]: 1.57; 95% CI: 1.10 to 2.24; *p* = 0.01) and walking alone (1.52; 95% CI: 1.14 to 2.03; *p* = 0.004) as compared to IFA, but there was no effect on time to achievement of other motor milestones. A limitation of our study is that we assessed child development up to 2 years of age, and, therefore, we may have not captured effects that are easier to detect or emerge at older ages.

**Conclusions:**

There was no benefit of prenatal MMS on child development outcomes up to 2 years of age as compared to IFA. There was evidence of an apparent positive effect of prenatal LNS on cognitive development trajectory and time to achievement of selected gross motor milestones.

**Trial registration:**

ClinicalTrials.gov NCT02145000.

## Abstract 2

Christopher R. Sudfeld and colleagues evaluate the benefit of multiple micronutrient supplementation and medium‐quantity lipid‐based nutrient supplementation in pregnancy on child development in rural Niger.

## Author summary

**Why was this study done?:**

- It is estimated that over 250 million children under 5 years of age do not reach their developmental potential in low- and middle-income country (LMIC) settings.
- Observational studies suggest that poor diet, anemia, and micronutrient deficiencies in pregnancy are associated with poor child development outcomes; however, the effect of macronutrient and micronutrient supplementation in pregnancy on child development outcomes remains inconclusive due to a limited number of randomized trials that have assessed developmental outcomes.

**What did the researchers do and find?:**

- We conducted a cluster-randomized controlled trial of prenatal multiple micronutrient supplementation (MMS) and lipid-based nutrient supplementation (LNS) as compared to routine iron–folic acid (IFA) supplementation among pregnant women in rural Niger and evaluated the effect on child development outcomes during the first 2 years of life.
- There was no benefit of prenatal MMS on child Bayley Scales of Infant and Toddler Development III (BSID-III) scores or on time to achievement of motor milestones as compared to IFA.
- There was no effect of prenatal LNS on BSID-III scores at 24 months of age as compared to IFA; however, the trajectory of child development differed between groups, with children in the prenatal LNS group having higher cognitive scores at 18 and 21 months of age as compared to IFA. Children whose mothers received prenatal LNS also had earlier achievement of sitting alone and walking alone as compared to children whose mothers received prenatal IFA.

**What do these findings mean?:**

- Prenatal supplementation with LNS appeared to support the achievement of selected motor milestones and improve cognitive development trajectory; however, there was no evidence of benefit of MMS over routine IFA on developmental outcomes in the context of rural Niger.
- Nevertheless, the apparent beneficial effects of prenatal LNS were limited, and, therefore, integrated interventions that consider maternal and child nutritional support along with parenting interventions and other components to support an enabling environment may provide larger effects on child development outcomes.

## Introduction

The first 1,000 days of life, the period from conception through 2 years of age, represents a critical window for child growth and brain development [1,2]. During this period, biological, environmental, and psychosocial exposures can lead to suboptimal cognitive, language, motor, and socioemotional development outcomes [3]. Suboptimal development during childhood may persist into adolescence and adulthood and lead to poor schooling achievement and decreased income and human capital [4,5]. Observational studies have reported that poor maternal diet, anemia, and low micronutrient status during pregnancy are associated with prematurity and fetal growth restriction [6,7], which, in turn, have been linked to poorer neurodevelopmental outcomes [8,9]. Undernutrition, poor weight gain in pregnancy, and micronutrient deficiencies are common among pregnant women in low- and middle-income countries (LMICs), and, therefore, supplementation to improve nutritional status in pregnancy may improve child development outcomes [10,11].

Randomized trials have evaluated the effect of multiple micronutrient supplementation (MMS) as compared to routine iron–folic acid (IFA) supplementation in pregnancy on a range of maternal and child outcomes in LMIC [12]. Prenatal MMS reduces the risk of low birthweight (LBW) and small-for-gestational age (SGA) births and may also reduce the risk of preterm birth as compared to IFA supplementation [12,13]. The effect of prenatal MMS on child development remains unclear with some trials finding marginal benefits of MMS on selected developmental domains while other trials have found no effect [14–19]. Prenatal lipid-based nutrient supplementation (LNS), which provides energy, protein, and essential fatty acids in addition to micronutrients, has been shown to have similar positive effects on birthweight and birth length [20]; however, evidence of the effect on child development outcomes from randomized trials is also mixed [16,17,20,21]. Therefore, while there is clear evidence that prenatal MMS and LNS can improve birth outcomes in LMICs, the evidence remains inconclusive for child development outcomes.

The objective of this study was to assess the effect of prenatal MMS and LNS on child development outcomes as compared to routine prenatal IFA supplementation. We analyzed data from a 3-arm cluster-randomized controlled trial of prenatal nutritional supplements in rural Niger that assessed child development with the Bayley Scales of Infant and Toddler Development III (BSID-III) and WHO gross motor milestones during the first 2 years of life as secondary outcomes.

## Methods

### Study design

A randomized, double-blind, placebo-controlled trial to assess the safety and efficacy of Rotasiil (Serum Institute of India), a live, oral rotavirus vaccine for infants, was conducted in Madarounda, Niger (clinicaltrials.gov identifier: NCT02145000) [22,23]. The rotavirus vaccine trial protocol and the primary results and adverse events have been published elsewhere [22]. Given the low immunogenicity of oral vaccines in high mortality settings, a nested cluster-randomized controlled trial of nutritional interventions in pregnancy (referred hereafter as the nutrition-immunogenicity trial) was conducted to evaluate whether prenatal MMS and LNS can increase the immunogenicity of rotavirus vaccine as compared to prenatal IFA, which is standard of care in Niger and most LMIC settings [24]. The nutrition-immunogenicity trial protocol and the primary results and adverse events have been published elsewhere [23]. The nutrition-immunogenicity trial was conducted concurrently with the rotavirus vaccine trial; pregnant women were first enrolled in the nutrition-immunogenicity trial and then infants of mothers who were enrolled in the nutrition-immunogenicity trial were screened at 6 to 8 weeks of age for enrollment in the rotavirus vaccine trial. We have previously reported that there was no difference in the primary outcome of infant immune response to rotavirus vaccine between prenatal LNS, MMS, and IFA supplementation [24]. In this study, we present an analysis of the nutrition-immunogenicity trial to assess the effect of prenatal MMS and LNS as compared to prenatal IFA on the secondary outcome of child development up to 2 years of age. Pregnant women were enrolled in the nutrition-immunogenicity trial from March 2015 and March 2016, and the last child 2-year follow-up visit was conducted in August 2019. There were no important changes to the nutrition-immunogenicity trial after enrollment commenced. This study is reported as per the “CONSORT extension for Cluster Trials” guideline (see S1 CONSORT Checklist).

Briefly, the nutrition-immunogenicity trial randomized 53 village clusters in a 1:1:1 allocation scheme to 1 of 3 prenatal supplementation groups: IFA (routine care), MMS, or LNS [24]. Randomization was performed by having the head of each village cluster select a piece of paper indicating the randomization group from an opaque jar. Villages were stratified by population size: <100; 100 to 249; ≥250 nonpregnant women of reproductive age. A total of 17 villages were randomized to IFA, 18 villages to MMS, and 18 villages to LNS.

Women of reproductive age in participating villages provided written informed consent for community-based monthly pregnancy surveillance, which included an at-home urine pregnancy test. Women with a confirmed pregnancy were then screened for trial enrollment at the health facility. The trial inclusion criteria were (i) <30 weeks gestation at the time of enrollment based on maternal report of the last menstrual period; (ii) intended to remain in the study area until 2 years postpartum; and (iii) did not have a chronic health condition, severe illness at screening, pregnancy complications (moderate to severe edema, hemoglobin (Hb) <7 g/dL, or diastolic blood pressure >90 mm Hg); and (iv) no self-reported peanut allergy [24]. Pregnant women who meet all inclusion criteria and provided written informed consent were enrolled in the nutrition-immunogenicity trial. A total of 3,332 pregnant women were enrolled in the nutrition-immunogenicity trial, of which 1,105 were in the IFA group, 1,083 in the MMS group, and 1,144 in the LNS group.

At 6 to 8 weeks after birth, infants born to women enrolled in the nutrition-immunogenicity trial were screened for enrollment in the randomized, double-blind, placebo-controlled vaccine trial of a live, oral rotavirus vaccine [22]. The inclusion criteria for infants in the rotavirus vaccine trial were (i) 6 to 8 weeks of age; (ii) able to swallow and have no history of vomiting within the past 24 hours; (iii) intended to remain in the study area for 2 years; and (iv) parent/guardian provided written informed consent. The analytic population for the current study consists of 2,551 children whose mothers completed prenatal supplementation in the nutrition-immunogenicity trial and were also enrolled in the rotavirus vaccine trial. There were 860 children in the IFA group, 777 children in the MMS group, and 874 children in the LNS group.

### Prenatal nutritional interventions

Pregnant women received nutritional supplements based on their village cluster from the time of randomization until delivery. The composition of the IFA, MMS, and LNS is detailed in the Table A in S1 Appendix. Pregnant women in the IFA standard of care group received tablets containing 60 mg iron and 400 μg folic acid (Remedica; Limassol, Cyprus). Pregnant women in the MMS group were provided capsules containing 30 mg iron, 400 μg folic acid, and 20 other micronutrients (DSM Nutritional Products; Isando, South Africa). Pregnant women in the LNS group received a 40-g fortified, ready-to-use food made of peanuts, oil, dried skimmed milk powder, and sugar (Nutriset S.A.S; Malaunay, France), which contained the same micronutrient content as the MMS. Based on nutritional composition, the LNS would be classified as a medium-quantity LNS [25]. Due to the inability to manufacture LNS completely indistinguishable from IFA tablets and MMS capsules, it was not possible to blind participants or field staff to their randomized group. The statistical analysis was conducted blinded to the randomized group using coded labels.

During pregnancy, home visits were conducted by research assistants every 7 days until delivery to distribute nutritional supplements. At each visit, the research assistants also obtained a count of the number of consumed nutritional supplements since the last visit. Adherence percentage for each participant was calculated as the total number of nutritional supplements consumed from enrollment to delivery as assessed at home visits divided by the expected total number of nutritional supplements the woman should have consumed from enrollment to delivery.

### Data collection and child development assessment

At the time of enrollment in the nutrition-immunogenicity trial, study midwives administered a standardized questionnaire to pregnant women, which assessed maternal and household sociodemographic characteristics and conducted a physical exam, and assessed maternal anthropometry (height, weight, and mid-upper arm circumference (MUAC)). Maternal Hb concentration was assessed from a finger-prick blood sample (Hemocue Hb 301, Angelholm, Sweden), and pregnant women received a malaria rapid diagnostic test (Biolin Malaria Ag *Pf* (HRP-2), Abbott Diagnostics, Scarborough, USA). Food security was assessed using the household hunger scale [26]. Improved sanitation was defined as a household having access to a flush toilet, improved pit latrine, or slab latrine. Improved water source was defined as households using covered or protected ground well for drinking water. Infants were screened for enrollment in the vaccine trial at 6 to 8 weeks of age. Community health assistants conducted monthly home visits from 3 to 24 months of age to assess child morbidity and growth.

A culturally adapted BSID-III with the cognitive, language, and motor scales was administered at the health facility at 6 months of age and every 3 months thereafter until 24 months of age [27]. To ensure cultural appropriateness, BSID-III items were adapted based on the consensus of a panel of local health staff and a child development expert. Adaptations included the replacement of unfamiliar images or terminology with more culturally relevant stimuli (e.g., changing a picture of an apple to a mango or using a simple wooden doll as are commonly found in villages of the study area). To maintain functional equivalence, replacement stimuli were selected to be of similar size, style, and complexity to the original stimuli. Eight female research nurses were selected and trained as dedicated BSID-III data collectors. Recruitment for the BSID-III assessment cohort was stopped on September 17, 2017, primarily due to resource constraints; the cohort of infants who turned 6 months of age after this date did not receive BSID-III assessments. Field-based supervision and weekly staff meetings were used to prevent assessor drift and ensure the continued quality of implementation. The BSID-III was administered in quiet and dedicated evaluation rooms at each health facility. The BSID-III showed high internal consistency for all domains (Cronbach’s alphas ≥0.85) (Table B in S1 Appendix). In addition, data collectors assessed maternal reports of achievement of the 6 WHO gross motor milestones every 4 weeks from 4 to 24 months of age: sitting without support, standing with assistance, hands-and-knees crawling, walking with assistance, standing alone, walking alone [28]. There were no changes to the developmental outcomes after the trial commenced.

### Sample size

The nutrition-immunogenicity trial sample size was based on the primary infant immunogenicity endpoint of seroconversion at 28 days post-oral rotavirus vaccine dose 3 assuming 90% power and a 20% absolute difference in the proportion of children that seroconvert between the randomized vaccine and placebo groups [23].

### Statistical analysis

The intention-to-treat (ITT) principle was used for all analyses and all analyses accounted for clustering by the village. Generalized linear regression models with cluster-robust standard errors were used to assess standardized mean differences (z-score differences) in BSID-III cognitive, language, and motor scores at 24 months of age (endline visit) using study-specific z-scores that were calculated from internal means and standard deviations among the full child population. We also assessed differences in BSID-III composite scores (US norms) for the 24-month visit by randomized groups [29,30]. We analyzed differences in the longitudinal trajectory of BSID-III cognitive, language, and motor raw scores from 6 to 24 months of age between randomized treatment arms with generalized linear mixed models (GLMMs). Multilevel models were constructed with the GLIMMIX procedure in SAS version 9.3 to take into account correlation within village clusters and correlation within children who were nested within the village clusters with random intercepts. GLMMs included randomized treatment arm, child age at assessment (6-, 9-, 12-, 15-, 18-, 21-, and 24-month time bins), an interaction term between treatment arm and infant age, and used a compound symmetry structure for within-subject correlation. If the overall test for difference in the trajectory of development domain scores between randomized groups was statistically significant, then differences in mean scores between groups at each child age were assessed using least-square means with Tukey–Kramer adjustment for multiple comparisons. In addition, Cox proportional hazard models with cluster-robust standard errors were used to assess the time to acquisition of each of the 6 WHO gross motor milestones; hazard ratios (HRs) >1.0 indicated the earlier achievement of the milestones (beneficial effect). Interaction terms between randomized group and time were used to assess the proportional hazards assumption.

As a sensitivity analysis to address the potential for baseline imbalance between randomized groups, we constructed multivariate models for all outcomes that included baseline covariates that may be associated with development outcomes based on a literature review including household wealth quintile, household size, food security, maternal age, maternal education, maternal anemia (Hb <11 g/dL), maternal underweight (body mass index (BMI) <18.5 kg/m^2^), malaria infection, the season of enrollment and child age and sex. Missingness for baseline variables was <5%, and missing indicators were used to retain participants in multivariable models. Further, we assessed potential bias due to dependent censoring (missing outcome data) on BSID-III scores at 24 months using inverse probability of censoring weights (IPCW) [31]. Stabilized censoring weights were constructed in models that included household wealth quintile, household size, food security, maternal age, maternal education, maternal anemia, maternal underweight, maternal malaria, and season of enrollment in the trial. Missing indicators were used to retain all participants in the calculation of censoring weights. In addition, we conducted exploratory analyses that assessed potential differences in the magnitude of the effect (effect modification) of MMS and LNS on BSID-III domain scores at 24 months of age as compared to IFA by baseline maternal education, maternal anemia, maternal underweight, and child sex. The effect modifiers were selected based on evidence that the effect of MMS on birth outcomes and child mortality differs by maternal nutritional status and child sex [13]. The Wald test was used to assess the statistical significance of interaction. Analyses were conducted in Stata Version 16 and SAS version 9.3.

### Ethics

The trial received ethical approval from the Comité Consultatif National d’Ethique in Niger, the Comité de Protection des Personnes in France, the Commission d’Ethique de la Recherche sur l’Etre Humain, Hôpitaux Universitaires de Genève in Switzerland, the Research Ethics Review Committee of the World Health Organization in Switzerland, and the Western Institutional Review Board in Olympia, WA. The trial was overseen by an independent Data and Safety Monitoring Board.

## Results

**Fig 1:**
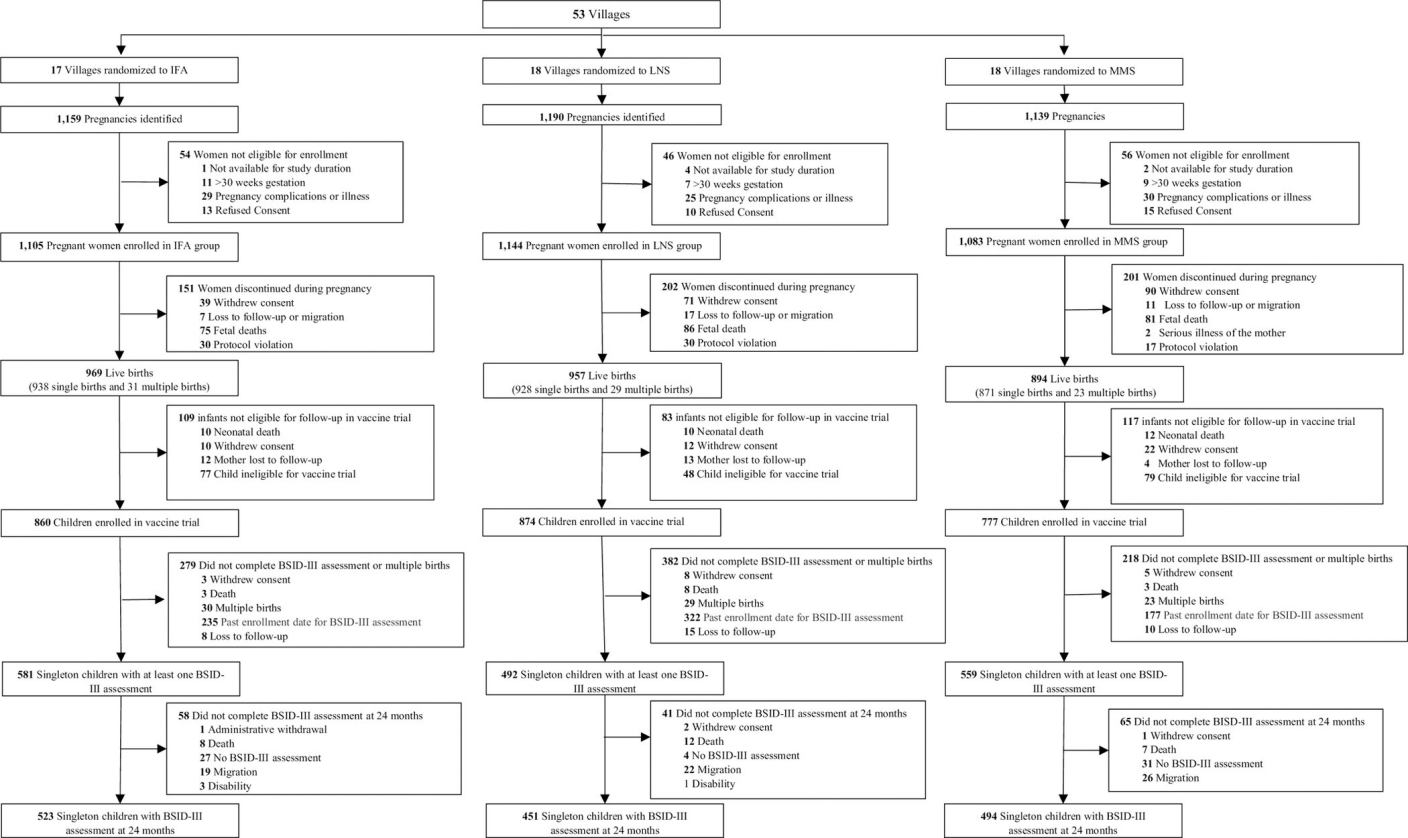
CONSORT flowchart for BSID-III assessments. BSID-III, Bayley Scales of Infant and Toddler Development III; IFA, iron–folic acid supplements; LNS, lipid-based nutrient supplements; MMS, multiple micronutrient supplements; WHO, World Health Organization.

**Fig 2:**
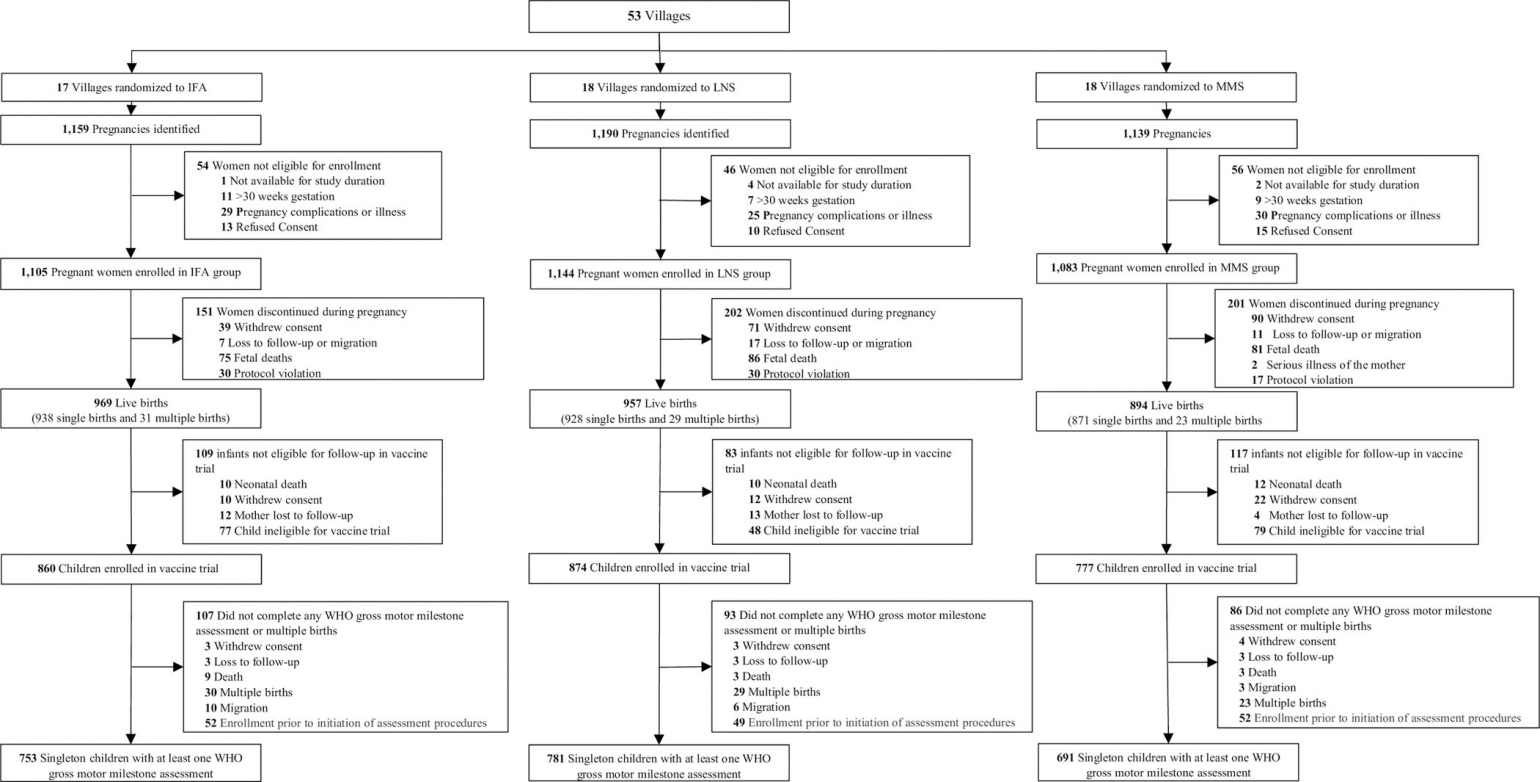
CONSORT flowchart for WHO motor milestone assessments. BSID-III, Bayley Scales of Infant and Toddler Development III; IFA, iron–folic acid supplements; LNS, lipid-based nutrient supplements; MMS, multiple micronutrient supplements; WHO, World Health Organization.

Pregnant women were enrolled and randomized in the nutrition-immunogenicity trial between March 2015 and March 2016, and the last child development follow-up visit was completed in August 2019. The baseline characteristics of the 3,332 pregnant women enrolled in the trial were similar across randomized groups (Table 1). The trial flow diagrams for the children included in the BSID-III and WHO gross motor milestone analyses are presented in Figs 1 and 2, respectively. There were 1,632 children who had at least 1 BSID-III assessment and 2,225 children with at least 1 WHO motor milestone assessment. Baseline characteristics for women whose children had an assessment as compared to women whose children did not have an assessment are presented for BSID-III and WHO motor milestones in Tables C and D in S1 Appendix, respectively. Women whose children had at least 1 BSID-III assessment had on average larger households and had a greater number of household assets as compared to women that did not have a BSID-III assessment. Women whose children had a WHO motor milestone assessment also tended to have large households, had a greater number of household assets, had less hunger, but had a lower proportion of women who completed primary school as compared to women that did not have a BSID-III assessment. Maternal adherence to the randomized regimen was high; the median adherence (Q1, Q3) to the supplementation regimen was 84% (70%, 93%) in the IFA arm, 86% (73%, 93%) in the MMS arm, and 88% (76%, 94%) in the LNS arm.

**Table 1:** Characteristics of pregnant women and children by randomized group.

	IFA Mean ± SD or n (%)	MMS Mean ± SD or n (%)	LNS Mean ± SD or n (%)
*Household characteristics*			
N	1,105	1,044	1,083
Household size, people	10.5 ± 6.8	10.0 ± 6.5	10.1 ± 6.6
Number of children <5 years	2.2 ± 1.9	2.4 ± 2.0	2.5 ± 1.9
Access to an improved latrine	694 (63.3)	605 (56.2)	585 (51.3)
Access to an improved water source	258 (23.4)	267 (24.7)	312 (27.3)
Little-to-no hunger in the past month	974 (88.4)	985 (91.2)	1,091 (95.8)
*Maternal characteristics*			
N	1,105	1,044	1,083
Age, years	26.5 ± 6.8	26.8 ± 6.8	26.9 ± 7.2
Married or cohabitating	1,081 (97.9)	1,068 (98.6)	1,128 (98.6)
Completed primary or higher education (≥6 years)	61 (5.5)	80 (7.4)	66 (5.8)
Underweight (BMI <18.5 kg/m^2^)	39 (3.9)	37 (3.7)	62 (5.9)
Anemic (Hb < 11 g/dL)	320 (32.5)	317 (32.7)	397 (38.4)
*Child characteristics*			
N	860	777	874
Randomized to Rotasiil vaccine	427 (49.7)	386 (49.7)	533 (61.0)
Randomized to placebo	433 (50.4)	391 (50.3)	341 (39.0)

BMI, body mass index; Hb, hemoglobin; IFA, iron–folic acid supplements; LNS, lipid-based nutrient supplements; MMS, multiple micronutrient supplements; SD, standard deviation.

**Fig 3:**
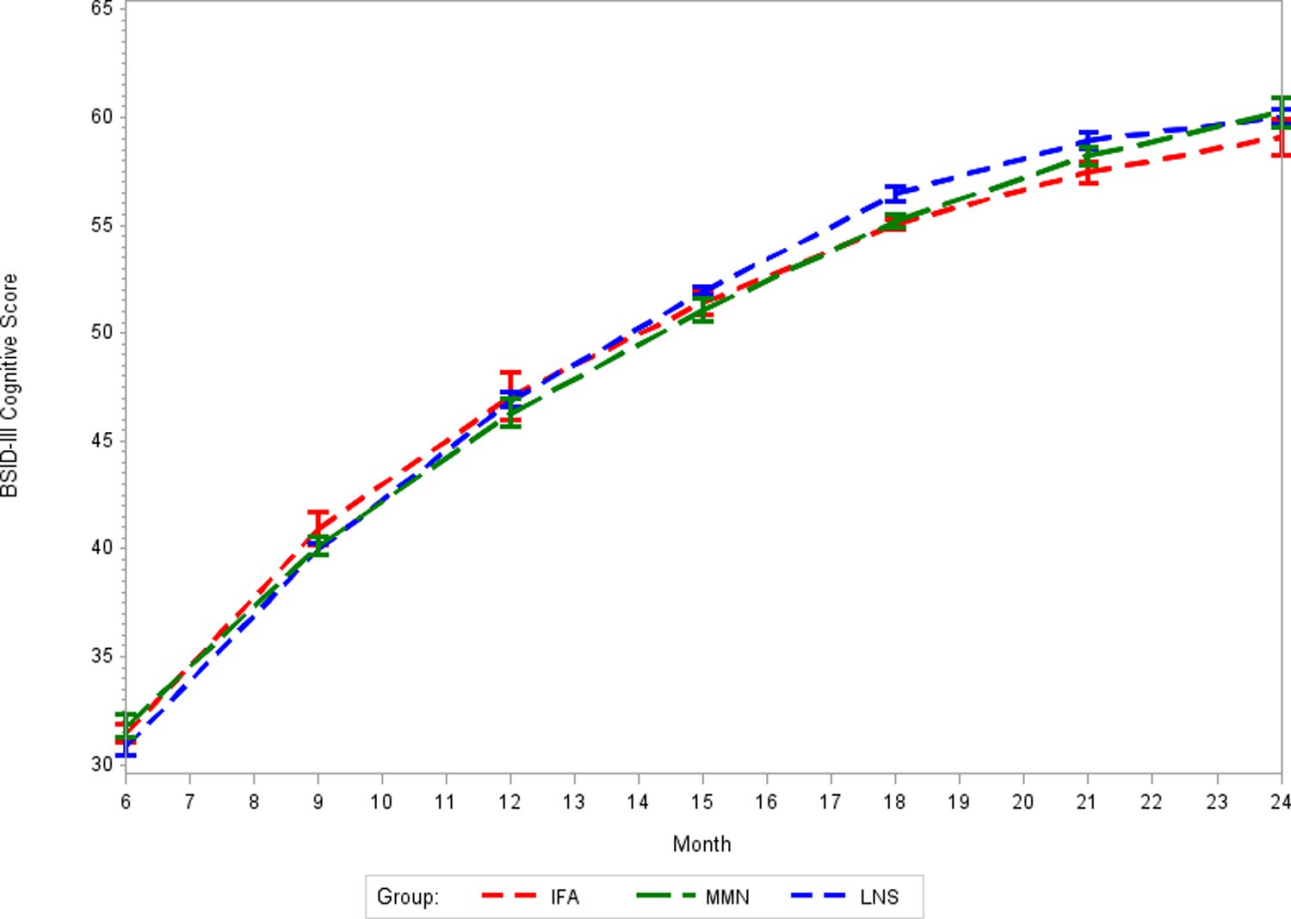
Mean BSID-III cognitive scores from 6 to 24 months for MMS, LNS, and IFA groups. *p*-Value for difference in trajectory: MMS vs. IFA = 0.28; LNS vs. IFA < 0.001. BSID-III, Bayley Scales of Infant and Toddler Development III; IFA, iron–folic acid supplements; LNS, lipid-based nutrient supplements; MMS, multiple micronutrient supplements.

**Fig 4:**
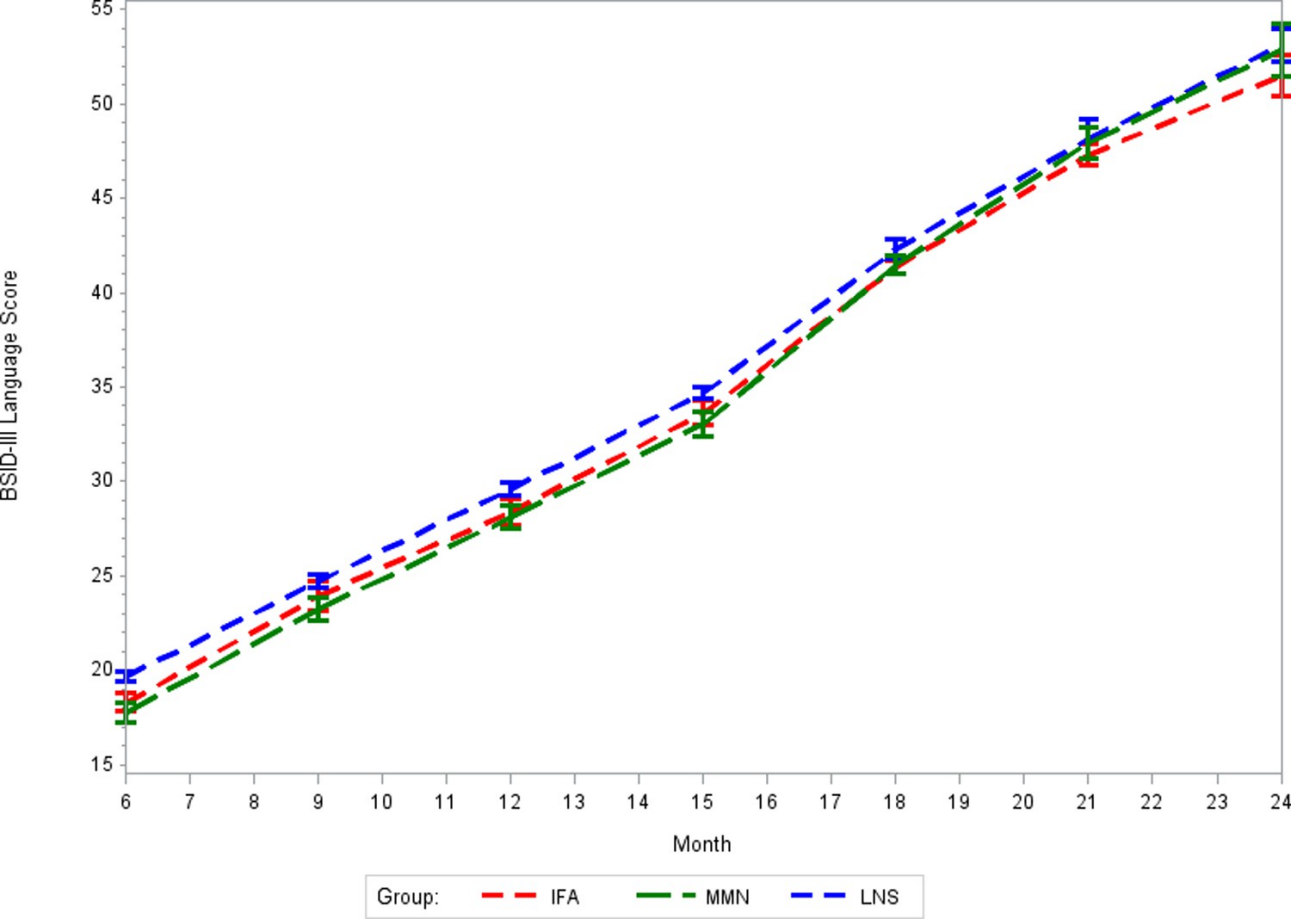
Mean BSID-III language scores from 6 to 24 months for MMS, LNS, and IFA groups. *p*-Value for difference in trajectory: MMS vs. IFA = 0.87; LNS vs. IFA = 0.60. BSID-III, Bayley Scales of Infant and Toddler Development III; IFA, iron–folic acid supplements; LNS, lipid-based nutrient supplements; MMS, multiple micronutrient supplements.

**Fig 5:**
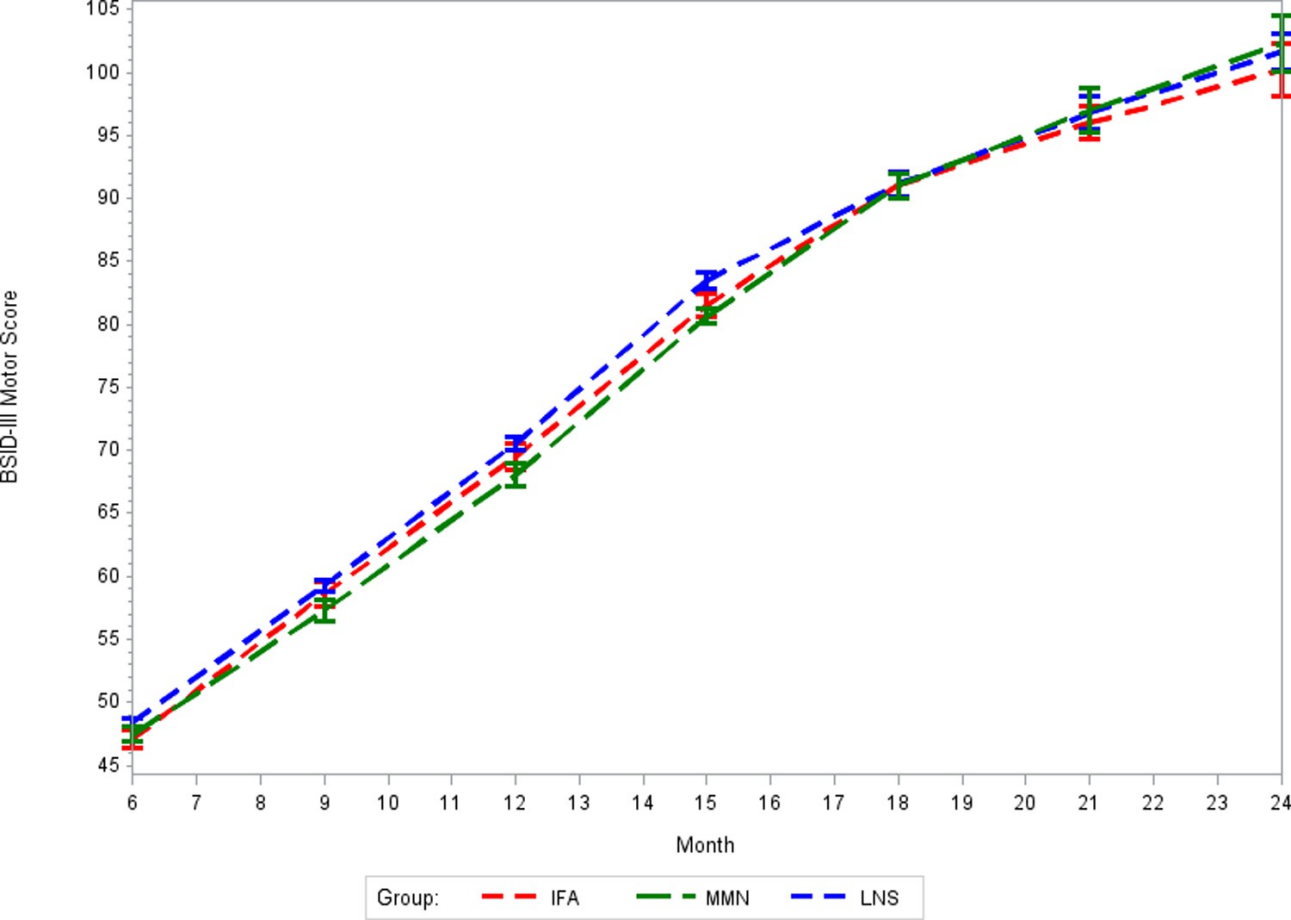
Mean BSID-III motor scores from 6 to 24 months for MMS, LNS, and IFA groups. *p*-Value for difference in trajectory: MMS vs. IFA = 0.10; LNS vs. IFA = 0.11. BSID-III, Bayley Scales of Infant and Toddler Development III; IFA, iron–folic acid supplements; LNS, lipid-based nutrient supplements; MMS, multiple micronutrient supplements.

There was no effect of prenatal MMS on BSID-III cognitive, language, or motor z-scores at 24 months of age as compared to routine prenatal IFA supplementation (*p*-values >0.05, Table 2). Correspondingly, there was no effect of prenatal MMS on BSID-III composite scores at 24 months of age as compared to IFA (Table E in S1 Appendix). The intracluster correlation coefficients (ICCs) for BSID-III domain scores at 24 months of age were 0.18 for cognitive scores, 0.22 for language scores, and 0.38 for motor scores. There was no difference in the effect of MMS on BSID-III domain scores at 24 months of age in sensitivity analyses that used multivariable models to account for potential baseline imbalances between randomized groups (Table F in S1 Appendix). In addition, there was no meaningful difference in the effect of MMS on BSID-III domain scores in sensitivity analyses using IPCW to account for missing outcome data (Table G in S1 Appendix). In exploratory analyses of potential effect modifiers (Table H in S1 Appendix), there was evidence of a potentially more positive effect of MMS compared to IFA on cognitive scores at 24 months of age among children whose mothers were not underweight at enrollment in pregnancy than among children whose mothers were underweight at enrollment (*p*-value for interaction: 0.03). There was also evidence of a potentially greater beneficial effect of MMS as compared to IFA on cognitive scores at 24 months of age for children whose mothers were nonanemic at enrollment in pregnancy as compared to children whose mothers were anemic at enrollment (*p*-value for interaction 0.01). There was no difference in cognitive, language, or motor score trajectories from 6 to 24 months between the MMS and IFA groups (Figs 3, 4, and 5, respectively). There was also no effect of prenatal MMS on time to achievement of any of the 6 gross motor milestones as compared to IFA (Table 3, survival plots presented in Figs A-F in S1 Appendix). There was also no effect on time to achievement of motor milestones in sensitivity analyses using multivariable models (Table I in S1 Appendix).

**Table 2:** Effect of randomized nutritional supplement group on BSID-III domain scores at 24 months of age.

	IFA Standardized Mean ± SD (*N* = 523)	MMS Standardized Mean ± SD (*N* = 494)	LNS Standardized Mean ± SD (*N* = 451)	MMS vs. IFA Mean Difference (95% CI)	*p*-value	LNS vs. IFA Mean Difference (95% CI)	*p*-value
*BSID-III domain z-scores*							
Cognitive	−0.11 ± 0.84	0.10 ± 0.99	0.06 ± 0.76	0.21 (−0.20, 0.62)	0.32	0.17 (−0.15, 0.49)	0.29
Language	−0.08 ± 0.89	0.08 ± 0.99	0.03 ± 0.92	0.16 (−0.30, 0.61)	0.50	0.11 (−0.22, 0.44)	0.53
Motor	0.00 ± 0.89	0.17 ± 0.95	−0.04 ± 0.75	0.18 (−0.39, 0.74)	0.54	−0.04 (−0.46, 0.38)	0.85

BSID-III, Bayley Scales of Infant and Toddler Development III; IFA, iron–folic acid supplements; LNS, lipid-based nutrient supplements; MMS, multiple micronutrient supplements; SD, standard deviation.

**Table 3:** Effect of MMS and LNS on time to achievement of the WHO gross motor milestones as compared to IFA.

	WHO Multicenter Growth Reference Study Median (IQR) age in months	IFA (*N* = 753) Median (IQR)	MMN (*N* = 691) Median (IQR)	LNS (*N* = 781) Median (IQR)	MMS vs. IFA HR for achievement (95% CI)	*p*-value	LNS vs. IFA HR for achievement (95% CI)	*p*-value
Sitting without support	5.9 (5.2, 6.7)	6.4 (5.5, 11.0)	5.5 (4.6, 7.4)	5.5 (4.6, 6.4)	1.18 (0.68, 2.04)	0.56	1.42 (0.87, 2.31)	0.16
Standing with assistance	7.4 (6.6, 8.4)	8.3 (7.4, 12.9)	8.3 (7.4, 10.1)	8.3 (7.4, 9.2)	1.11 (0.64, 1.93)	0.85	1.42 (0.90, 2.26)	0.15
Hands-and-knees crawling	8.3 (7.4, 9.3)	9.2 (8.3, 13.8)	9.2 (8.3, 12.0)	9.2 (7.4, 10.1)	1.05 (0.61, 1.80)	0.72	1.42 (0.88, 2.28)	0.14
Walking with assistance	9.0 (8.2, 10.0)	12.0 (10.1, 14.7)	12.0 (10.1, 14.7)	10.1 (9.2, 12.9)	0.99 (0.61, 1.61)	0.97	1.43 (0.94, 2.19)	0.10
Standing alone	10.8 (9.7, 12.0)	13.8 (12.0, 16.6)	13.8 (11.0, 16.6)	12.0 (10.1, 13.8)	1.08 (0.69, 1.69)	0.75	1.57 (1.10, 2.24)	0.01
Walking alone	12.0 (11.0, 13.0)	15.6 (12.9, 17.5)	14.7 (12.9, 17.5)	13.8 (12.0, 15.6)	1.04 (0.71, 1.54)	0.83	1.52 (1.14, 2.03)	0.004

HRs >1.0 indicate earlier achievement (beneficial effect).

HR, hazard ratio; IFA, iron-folic acid supplements; IQR, interquartile range; LNS, lipid-based nutrient supplements; MMS, multiple micronutrient supplements; WHO, World Health Organization.

There was no effect of prenatal LNS on BSID-III cognitive, language, or motor z-scores at 24 months of age as compared to routine prenatal IFA supplementation (*p*-values >0.05, Table 2). There was also no effect of LNS on BSID-III composite scores at 24 months of age as compacted to IFA (Table E in S1 Appendix). There was no difference in the effect of LNS on BSID-III domain scores at 24 months in a sensitivity analysis that used multivariable models to account for potential baseline imbalances between randomized groups (Table F in S1 Appendix). In addition, there was no meaningful difference in the effect of LNS on BSID-III domain scores in sensitivity analyses using IPCW to account for missing outcome data (Table G in S1 Appendix). In exploratory analyses of effect modifiers (Table H in S1 Appendix), there was evidence that LNS may provide a greater positive effect on BSID-III motor scores at 24 months of age compared to IFA for children whose mothers were not anemic at enrollment in pregnancy as compared to children whose mothers were anemic at enrollment (*p*-value for interaction 0.03). The trajectory of cognitive scores from 6 to 24 months of age differed for children whose mothers were randomized to the LNS group as compared to the IFA group (Fig 3: *p*-value for difference in trajectory <0.001). The LNS group had significantly higher cognitive scores at 18 and 21 months as compared to IFA (Table J in S1 Appendix), and the magnitude of the difference was relatively large at approximately 1.4 points, which equated to approximately 0.35 SD at each time point. There was no difference in BSID-III language and motor score trajectories from 6 to 24 months of age for the LNS as compared to the IFA group (Figs 4 and 5, respectively, *p*-values >0.05). Children whose mothers received LNS had significantly earlier achievement of sitting alone and walking alone as compared to IFA (*p*-values <0.05), but there was no difference between prenatal LNS and IFA groups in time to achievement of sitting without support, standing with assistance, hands-and-knees crawling or walking with assistance (Table 3, survival plots presented in Figs A-F in S1 Appendix). The findings of the effect of LNS on time to achievement of motor milestones were consistent in sensitivity analyses using multivariable models (Table I in S1 Appendix).

## Discussion

In this cluster-randomized trial conducted in rural Niger, we evaluated the effect of prenatal MMS and LNS as compared to routine prenatal IFA supplementation on child BSID-III domain scores and time to achievement of the WHO gross motor milestones. There was no evidence of an effect of MMS on child cognitive, language, or motor BSID-III scores at 24 months of age, BSID-III score trajectories from 6 to 24 months of age, or time to achievement of WHO gross motor milestones as compared to routine prenatal IFA. Prenatal LNS did not affect child cognitive, language, or motor BSID-III scores at 24 months of age as compared to IFA. However, children in the prenatal LNS group had a different trajectory of BSID-III cognitive scores from 6 to 24 months of age with higher scores at 18 and 21 months of age as compared to children in the prenatal IFA group. Children in the prenatal LNS group also had earlier achievement of the WHO milestones of sitting alone and walking alone as compared to children in the IFA group.

We found no evidence that prenatal MMS provided benefits for any child development outcome as compared to routine prenatal IFA supplementation. Randomized trials have consistently indicated that MMS reduces the risk of LBW and SGA births, which, in turn, are associated with suboptimal development outcomes in observational studies [9,12,13]. Despite the potential for benefit, the evidence on the direct effect of MMS in pregnancy on development outcomes in randomized trials is mixed [16,17,20,21]. An MMS trial in Indonesia found that children of mothers who received MMS had higher procedural memory at 9 to 12 years of age as compared to IFA and there was also a greater beneficial effect of MMS on general intelligence scores among children whose mothers were anemic at baseline [15]. In contrast to the subgroup findings in Indonesia, we found that the effect of MMS on cognitive scores may be greater for non-underweight and nonanemic women in the setting of rural Niger. Further, a study in rural China also found positive effects of MMS on intelligence quotient (IQ) and verbal comprehension scores at 14 years of age; however, several MMS trials in Nepal, Ghana, Malawi, and Tanzania have found no overall effect on development outcomes at variable ages [14,16–19]. Overall, the available evidence, including the results from our trial, does not consistently show beneficial effects of MMS on child development outcomes. Nevertheless, there is consistent evidence that MMS reduces the risk of adverse birth outcomes, and, therefore, routine MMS should continue to be considered for implementation as a public health program in LMIC [12,13,32,33].

We found that prenatal LNS supplementation, which provides macronutrients in addition to micronutrients, had apparent beneficial effects on child cognitive development trajectory at 18 and 21 months as compared to routine prenatal IFA, although there were no sustained effects on cognitive scores at 24 months of age. We also found evidence of positive effects of prenatal LNS as compared to IFA on achievement of sitting and walking alone. To date, only 3 trials have assessed the effect of maternal LNS on child development outcomes [16,17,21]. The International Lipid-Based Nutrient Supplements (iLiNS) trials conducted in Ghana and Malawi found that small-quantity lipid nutrient supplements (SQ-LNS) provided to mothers in pregnancy and up to 6 months postpartum in combination with child SQ-LNS from 6 to 18 months of age had positive effects on time to achievement of children walking alone, but there was no effect on child motor, language, socioemotional development, or executive function at 18 months of age, as compared to routine IFA supplementation [16,17]. However, due to the iLiNS study design, it is not clear whether the positive effect on walking was attributable to maternal or child SQ-LNS supplementation. In addition, a trial in Madagascar found no effect of combined maternal and child LNS on child motor, problem-solving, communication, or socioemotional development [21]. As a result, prenatal LNS may support the achievement of selected motor milestones, but the available evidence on the effect on other development domains remains inconclusive.

Our study has important strengths and limitations. First, a major strength of our study was the repeated assessments of BSID-III, which allowed us to examine differences in development trajectories that enabled us to detect positive effects in cognitive development at 18 to 21 months for LNS. Cognitive trajectories during the first 2 years of life, rather than single time point assessments, have been shown to importantly differentiate middle-childhood and adolescence cognitive function [34]. Second, our study was conducted in rural Niger, which provides much-needed evidence in the context of a population of mothers and children at high risk for undernutrition and a population that has limited data on child development. In terms of limitations, we assessed child development to 2 years of age and therefore may not have captured beneficial effects that may be easier to detect or emerge at older child ages. For example, the positive effects in MMS trials in Indonesia and China were detected in mid-childhood and early adolescence [14,15]. In addition, the use of the BSID-III in the context of rural Niger, despite care in adaptation to the setting, may result in some degree of nondifferential misclassification of children’s developmental abilities across randomized groups if some BSID-III items were not fully capturing children’s cognitive, language, motor development and would therefore bias results to the null. Further, not all infants enrolled in the trial completed child development assessments, and, therefore, bias due to loss to follow-up is possible; however, there was no meaningful difference between the results of the IPCW sensitivity analyses and the primary analyses, which suggests that there is limited potential for bias due to dependent censoring. Last, we analyzed multiple secondary trial outcomes, and, therefore, we cannot rule out that our findings of a beneficial effect of prenatal LNS on cognitive development trajectory and achievement of sitting alone and walking alone were not due to type I errors (incorrectly rejecting the null) due to multiple testing.

Overall, we found that prenatal MMS did not improve child development outcomes as compared to routine prenatal IFA, while prenatal LNS appeared to provide beneficial effects on cognitive development trajectory and achievement of selected gross motor scores as compared to IFA. The positive findings for LNS on selected outcomes, but not MMS, in our trial suggest that strategies to improve maternal diets and combined macronutrient and micronutrient supplementation may have a greater potential to provide child development benefits as compared to micronutrient supplementation alone. However, the lack of an effect at 24 months for LNS as compared to IFA also suggests that nutritional support in pregnancy alone may not support sustained improvements in child development outcomes. Research and programs should consider integrated interventions that go beyond nutrition alone and more broadly promote nurturing care through the inclusion of interventions that also support health, security, safety, responsive caregiving, and opportunities for early learning [35].

## Supporting information

S1 CONSORT Checklist CONSORT extension checklist for cluster trials. (DOCX)

S1 Appendix Appendix tables and figures. Table A in S1 Appendix. Nutrient content of nutritional supplements. Table B in S1 Appendix. Internal consistency as measured by Cronbach’s alpha for BSID-III raw domain scores. Table C in S1 Appendix. Baseline characteristics of pregnant women whose child has a BSID-III assessment as compared to pregnant women whose child did not have a BSID-III assessment. Table D in S1 Appendix. Baseline characteristics of pregnant women whose child has a WHO motor milestone assessment as compared to pregnant women whose child did not have a WHO motor milestone assessment. Table E in S1 Appendix. Effect of MMS and LNS on BSID-III composite scores using US norms at 24 months of age as compared to IFA. The composite mean score is 100.0 and standard deviation of 15.0. Table F in S1 Appendix. Multivariable analyses of the effect of MMS and LNS on BSID-III z-scores at 24 months of age as compared to IFA. Table G in S1 Appendix. IPCW analyses of the effect of MMS and LNS on BSID-III z-scores at 24 months of age as compared to IFA. Table H in S1 Appendix. Modifiers of the effect of MMS and LNS on BSID-III z-scores at 24 months of age. Table I in S1 Appendix. Multivariable analyses of the effect of MMS and LNS on time to achievement of the WHO gross motor milestones as compared to IFA. Table J in S1 Appendix. Modeled means and standard errors for BSID-III cognitive scores at 6, 9, 12, 15, 18, 21, and 24 months by for LNS versus IFA (*p*-value for difference in test of trajectory <0.001) Fig A in S1 Appendix. Survival plot for time to achievement of sitting without support stratified by randomized group. Fig B in S1 Appendix. Survival plot for time to achievement of standing with assistance stratified by randomized group. Fig C in S1 Appendix. Survival plot for time to achievement of hands-and-knees crawling stratified by randomized group. Fig D in S1 Appendix. Survival plot for time to achievement of walking with assistance stratified by randomized group. Fig E in S1 Appendix. Survival plot for time to achievement of standing alone stratified by randomized group. Fig F in S1 Appendix. Survival plot for time to achievement of walking alone stratified by randomized group. BSID-III, Bayley Scales of Infant and Toddler Development III; IFA, iron–folic acid; IPCW, inverse probability of censoring weights; LNS, lipid-based nutrient supplementation; MMS, multiple micronutrient supplementation.
(DOCX)

## Abbreviations

BMI: body mass index
BSID-III: Bayley Scales of Infant and Toddler Development III
GLMM: generalized linear mixed model
Hb: hemoglobin
HR: hazard ratio
ICC: intracluster correlation coefficient
IFA: iron–folic acid
iLiNS: International Lipid-Based Nutrient Supplements
IPCW: inverse probability of censoring weights
IQ: intelligence quotient
ITT: intention-to-treat
LBW: low birthweight
LMIC: low- and middle-income country
LNS: lipid-based nutrient supplementation
MMS: multiple micronutrient supplementation
MUAC: mid-upper arm circumference
SGA: small-for-gestational age
SMD: standardized mean difference
SQ-LNS: small-quantity lipid nutrient supplements
