# Supplementary material for: Evaluation of multiple micronutrient supplementation and medium-quantity lipid-based nutrient supplementation in pregnancy on child development in rural Niger: A secondary analysis of a cluster randomized controlled trial
Source: PLoS Med. 2022 May 2;19(5):e1003984. doi: 10.1371/journal.pmed.1003984 (PMC9060361; doi:10.1371/journal.pmed.1003984)
Supplement: S1 Appendix — Table A in S1 Appendix. Nutrient content of nutritional supplements. Table B in S1 Appendix. Internal consistency as measured by Cronbach’s alpha for BSID-III raw domain scores. Table C in S1 Appendix. Baseline characteristics of pregnant women whose child has a BSID-III assessment as compared to pregnant women whose child did not have a BSID-III assessment. Table D in S1 Appendix. Baseline characteristics of pregnant women whose child has a WHO motor milestone assessment as compared to pregnant women whose child did not have a WHO motor milestone assessment. Table E in S1 Appendix. Effect of MMS and LNS on BSID-III composite scores using US norms at 24 months of age as compared to IFA. The composite mean score is 100.0 and standard deviation of 15.0. Table F in S1 Appendix. Multivariable analyses of the effect of MMS and LNS on BSID-III z-scores at 24 months of age as compared to IFA. Table G in S1 Appendix. IPCW analyses of the effect of MMS and LNS on BSID-III z-scores at 24 months of age as compared to IFA. Table H in S1 Appendix. Modifiers of the effect of MMS and LNS on BSID-III z-scores at 24 months of age. Table I in S1 Appendix. Multivariable analyses of the effect of MMS and LNS on time to achievement of the WHO gross motor milestones as compared to IFA. Table J in S1 Appendix. Modeled means and standard errors for BSID-III cognitive scores at 6, 9, 12, 15, 18, 21, and 24 months by for LNS versus IFA (p-value for difference in test of trajectory <0.001) Fig A in S1 Appendix. Survival plot for time to achievement of sitting without support stratified by randomized group. Fig B in S1 Appendix. Survival plot for time to achievement of standing with assistance stratified by randomized group. Fig C in S1 Appendix. Survival plot for time to achievement of hands-and-knees crawling stratified by randomized group. Fig D in S1 Appendix. Survival plot for time to achievement of walking with assistance stratified by randomized group. Fig E in S1 Appendix. Survival plo [file pmed.1003984.s002.docx]

**Table A.** Nutrient content of nutritional supplements

|  | **US RDA/AI for**  **pregnant**  **women** | **Iron-Folic acid**  **(IFA)** | **Multiple Micronutrient Supplements (MMS)** | **Lipid Nutrient Supplements (40g)** |
| --- | --- | --- | --- | --- |
| Energy (kcal) | 2500 | .. | .. | 237 |
| Proteins (g) | 50 | .. | .. | 5.2 |
| Milk proteins (g) | .. | .. | .. | 1.7 |
| Lipids (g) | .. | .. | .. | 20 |
| LA (*Linoleic Acid*) (g) | .. | .. | .. | 6.9 |
| ALA (*α-Linolenic Acid*) (g) | .. | .. | .. | 1.16 |
| Calcium (mg) | 1000 | .. | 559 | 559 |
| Phosphorus (*free*) (mg) | 700 | .. | 400 | 400 |
| Potassium (mg) | 4700 | .. | 400 | 400 |
| Magnesium (mg) | 350 | .. | 130 | 130 |
| Zinc (mg) | 11 | .. | 30 | 30 |
| Copper (mg) | 1 | .. | 4 | 4 |
| Iron (mg) | 27 | 60 | 30 | 30 |
| Manganese (mg) | 2 | .. | 2.6 | 2.6 |
| Iodine (µg) | 220 | .. | 250 | 250 |
| Selenium (µg) | 60 | .. | 130 | 130 |
| Vitamin A (µg) | 800 | .. | 800 | 800 |
| Vitamin B_1_ (mg) | 1.4 | .. | 2.8 | 2.8 |
| Vitamin B_2_ (mg) | 1.4 | .. | 2.8 | 2.8 |
| Niacin B_3_ (mg) | 18 | .. | 36 | 36 |
| Pantothenic acid - B_5_(mg) | 6 | .. | 7 | 7 |
| Vitamin B_6_ (mg) | 1.9 | .. | 3.8 | 3.8 |
| Vitamin B_12_ (µg) | 2.6 | .. | 5.2 | 5.2 |
| Folic acid (µg) | 600 | 400 | 400 | 400 |
| Vitamin C (mg) | 85 | .. | 100 | 100 |
| Vitamin D_3_ (µg) | 15 | .. | 15 | 15 |
| Vitamin E (mg) | 15 | .. | 20 | 20 |
| Vitamin K_1_ (µg) | 90 | .. | 45 | 45 |

IFA: Iron-folic acid supplements

LNS: Lipid-based nutrient supplements

MMS: Multiple micronutrient supplements

US: United States

RDA: Recommended Dietary Allowance

AI: Adequate Intake

**Table B.** Internal consistency as measured by Cronbach’s alpha for Bayley Scales of Infant and Toddler Development Scores –III raw domain scores

|  | Internal consistency  Cronbach’s alpha |
| --- | --- |
| Cognitive scale | 0.85 |
|  |  |
| Language scale | 0.91 |
| Receptive language | 0.86 |
| Expressive language | 0.84 |
|  |  |
| Motor scale | 0.91 |
| Fine motor | 0.86 |
| Gross motor | 0.86 |

**Table C.** Baseline characteristics of pregnant women whose child has a BSID-III assessment as compared to pregnant women whose child did not have a BSID-III assessment.

|  | **Pregnant women with child that had a WHO motor milestone assessment** | **Pregnant women with child that *did not* have a WHO motor milestone assessment** | **p-value** |
| --- | --- | --- | --- |
| N | 1632 | 1709 |  |
| Household size, people | 10.7±7 | 9.7±6.3 | 0.003 |
| Number of children <5 years | 2.5±2 | 2.3±1.8 | 0.05 |
| Wealth index | 0.2±1.6 | -0.2±1.7 | 0.04 |
| Little-to-no hunger in the past month | 1630 (89.4) | 1701 (94.1) | 0.10 |
| Age, years | 26.7±6.9 | 26.8 ± 7 | 0.61 |
| Completed primary or higher education (≥6 years) | 163 (5.6) | 170.7 (6.7) | 0.19 |
| Underweight (BMI<18.5 kg/m^2^) | 64 (4.5) | 74 (4.6) | 0.91 |
| Anemic (Hb < 11 g/dL) | 466 (33.2) | 574 (36) | 0.32 |

IFA: Iron-folic acid supplements

LNS: Lipid-based nutrient supplements

MMS: Multiple micronutrient supplements

SD: Standard deviation

BMI: Body mass index

Hb: Hemoglobin

WHO: World Health Organization

**Table D.** Baseline characteristics of pregnant women whose child has a WHO motor milestone assessment as compared to pregnant women whose child did not have a WHO motor milestone assessment.

|  | **Pregnant women with child that had a BSID-III assessment** | **Pregnant women with child that *did not* have a BSID-III assessment** | **p-value** |
| --- | --- | --- | --- |
| N | 2225 | 1116 |  |
| Household size, people | 10.4±6.7 | 9.8±6.4 | 0.05 |
| Number of children <5 years | 2.4±1.9 | 2.3±1.9 | 0.06 |
| Wealth index | 0.1±1.7 | -0.2±1.7 | 0.02 |
| Little-to-no hunger in the past month | 2105 (94.8) | 953 (85.9) | 0.01 |
| Age, years | 26.9 ± 6.9 | 26.4 ± 6.9 | 0.12 |
| Completed primary or higher education (≥6 years) | 124 (5.6) | 83 (7.4) | 0.01 |
| Underweight (BMI<18.5 kg/m^2^) | 104 (4.8) | 34 (3.8) | 0.28 |
| Anemic (Hb < 11 g/dL) | 744 (34.9) | 291 (33.8) | 0.56 |

IFA: Iron-folic acid supplements

LNS: Lipid-based nutrient supplements

MMS: Multiple micronutrient supplements

SD: Standard deviation

BMI: Body mass index

Hb: Hemoglobin

**Table E.** Effect of MMS and LNS on BSID-III composite scores using US norms at 24 months of age as compared to IFA. The composite mean score is 100.0 and standard deviation of 15.0.

|  | **IFA Standardized Mean ± SD**  **(N=523)** | **MMS Standardized Mean ± SD**  **(N=494)** | **LNS Standardized Mean ± SD**  **(N=451)** | **MMS vs. IFA Mean Difference (95% CI)** | **LNS vs. IFA Mean Difference (95% CI)** |
| --- | --- | --- | --- | --- | --- |
| *BSID-III domain composite scores* |  |  |  |  |  |
| Cognitive | 93.6 ± 9.9 | 95.7 ± 12.0 | 95.0 ± 9.4 | 2.2 (-2.2, 6.5) | 1.4 (-2.0, 4.7) |
| Language | 94.4 ± 11.2 | 96.0 ± 12.1 | 95.6 ± 11.6 | 1.6 (-4.0, 7.2) | 1.2 (-3.1, 5.4) |
| Motor | 113.5 ± 20.8 | 117.6 ± 22.2 | 112.1 ± 18.7 | 4.1 (-9.6, 17.7) | -1.4 (-11.8, 9.0) |

BSID-III: Bayley Scales of Infant Development III

IFA: Iron-folic acid supplements

LNS: Lipid-based nutrient supplements

MMS: Multiple micronutrient supplements

SD: Standard deviation

**Table** **F.** Multivariable* analyses of the effect of MMS and LNS on BSID-III z-scores at 24 months of age as compared to IFA

|  | **IFA Standardized Mean ± SD**  **(N=523)** | **MMS Standardized Mean ± SD**  **(N=494)** | **LNS Standardized Mean ± SD**  **(N=451)** | **MMS vs. IFA Mean Difference (95% CI)** | **LNS vs. IFA Mean Difference (95% CI)** |
| --- | --- | --- | --- | --- | --- |
| *BSID-III domain z-scores* |  |  |  |  |  |
| Cognitive | -0.11 ± 0.84 | 0.10 ± 0.99 | 0.06 ± 0.76 | 0.13 (-0.12, 0.38) | 0.15 (-0.19, 0.50) |
| Language | -0.08 ± 0.89 | 0.08 ± 0.99 | 0.03 ± 0.92 | 0.03 (-0.25, 0.30) | 0.11 (-0.31, 0.52) |
| Motor | 0.00 ± 0.89 | 0.17 ± 0.95 | -0.04 ± 0.75 | -0.16 (-0.51, 0.19) | 0.12 (-0.39, 0.63) |
|  |  |  |  |  |  |

*Multivariable adjusted estimates control for household wealth quintile, household size, food security, maternal age (in years), education (any vs. none), anemia (Hb<11 g/dL vs. Hb ≥11 g/dL), underweight (BMI <18.5 kg/m2 vs. BMI ≥18.5 kg/m2), malaria (positive RDT vs. negative RDT), child age and sex, and season of enrollment in the trial (hunger vs. not hunger season). Missing indicators were used to retain participants. Models accounted for clustering at the village level.

BSID-III: Bayley Scales of Infant Development III

IFA: Iron-folic acid supplements

LNS: Lipid-based nutrient supplements

MMS: Multiple micronutrient supplements

SD: Standard deviation

**Table G.** Inverse probability of censoring weighted (IPCW)* analyses of the effect of MMS and LNS on BSID-III z-scores at 24 months of age as compared to IFA

|  | **MMS vs. IFA Mean Difference (95% CI)** | **LNS vs. IFA Mean Difference (95% CI)** |
| --- | --- | --- |
| *BSID-III domain z-scores at 24 months* |  |  |
| Cognitive | 0.17 (-0.16, 0.49) | 0.19 (-0.23, 0.60) |
| Language | 0.11 (-0.22, 0.45) | 0.15 (-0.29, 0.60) |
| Motor | -0.03 (-0.46, 0.40) | 0.17 (-0.39, 0.72) |
|  |  |  |

*Inverse probability weights calculated using household wealth quintile, household size, food security, maternal age (in years), maternal education (any, none, missing), anemia (Hb<11 g/dL vs. Hb ≥11 g/dL, missing), underweight (BMI <18.5 kg/m2, BMI ≥18.5 kg/m2, missing), malaria (positive RDT vs. negative RDT, missing), and season of enrollment in the trial (hunger, no hunger season, missing). Missing indicators were used to retain participants. Models accounted for clustering at the village level.

BSID-III: Bayley Scales of Infant Development III

IFA: Iron-folic acid supplements

LNS: Lipid-based nutrient supplements

MMS: Multiple micronutrient supplements

SD: Standard deviation

**Table H.** Modifiers of the effect of MMS and LNS on BSID-III z-scores at 24 months of age

|  | **Cognitive z-score** | | **Language z-score** | | **Motor z-score** | |
| --- | --- | --- | --- | --- | --- | --- |
|  | **MMS vs. IFA Standardized mean difference (95% CI)** | **LNS vs. IFA Standardized mean difference (95% CI)** | **MMS vs. IFA Standardized mean difference (95% CI)** | **LNS vs. IFA Standardized mean difference (95% CI)** | **MMS vs. IFA Standardized mean difference (95% CI)** | **LNS vs. IFA Standardized mean difference (95% CI)** |
| Maternal education |  |  |  |  |  |  |
| Mother did not complete primary school | 0.14 (-0.14, 0.41) | 0.15 (-0.21, 0.52) | 0.13 (-0.31, 0.57) | 0.12 (-0.39, 0.64) | -0.08 (-0.62, 0.46) | 0.14 (-0.48, 0.76) |
| Mother completed primary school | 0.22 (-0.18, 0.62) | 0.28 (-0.20, 0.76) | 0.04 (-0.18, 0.26) | 0.20 (-0.18, 0.57) | 0.00 (-0.23, 0.23) | 0.23 (-0.24, 0.69) |
| p-value for interaction | 0.57 | 0.30 | 0.64 | 0.62 | 0.68 | 0.54 |
|  |  |  |  |  |  |  |
| Maternal anemia status at baseline |  |  |  |  |  |  |
| Non-anemic (Hb ≥ 11 g/dL) | 0.26 (-0.06, 0.58) | 0.23 (-0.17, 0.64) | 0.13 (-0.20, 0.45) | 0.16 (-0.28, 0.59) | 0.00 (-0.37, 0.37) | 0.25 (-0.29, 0.79) |
| Anemic (Hb < 11 g/dL) | -0.05 (-0.39, 0.28) | 0.10 (-0.30, 0.50) | 0.02 (-0.34, 0.37) | 0.13 (-0.39, 0.64) | -0.20 (-0.74, 0.34) | -0.03 (-0.68, 0.62) |
| p-value for interaction | 0.01 | 0.18 | 0.18 | 0.73 | 0.08 | 0.03 |
|  |  |  |  |  |  |  |
| Maternal underweight status |  |  |  |  |  |  |
| Not underweight (BMI<18.5) | 0.19 (-0.13, 0.51) | 0.22 (-0.19, 0.64) | 0.10 (-0.23, 0.44) | 0.16 (-0.29, 0.61) | -0.03 (-0.44, 0.39) | 0.18 (-0.37, 0.73) |
| Underweight (BMI<18.5) | -0.21 (-0.59, 0.17) | -0.12 (-0.74, 0.50) | 0.18 (-0.28, 0.64) | 0.14 (-0.51, 0.78) | -0.29 (-1.05, 0.47) | 0.14 (-0.86, 1.14) |
| p-value for interaction | 0.03 | 0.26 | 0.71 | 0.91 | 0.36 | 0.92 |
|  |  |  |  |  |  |  |
| Child sex |  |  |  |  |  |  |
| Female | 0.20 (-0.17, 0.57) | 0.14 (-0.30, 0.58) | 0.12 (-0.28, 0.52) | 0.09 (-0.34, 0.52) | -0.02 (-0.54, 0.50) | 0.10 (-0.45, 0.65) |
| Male | 0.15 (-0.15, 0.45) | 0.28 (-0.13, 0.69) | 0.09 (-0.19, 0.38) | 0.23 (-0.30, 0.75) | -0.06 (-0.37, 0.26) | 0.26 (-0.36, 0.87) |
| p-value for interaction | 0.65 | 0.24 | 0.85 | 0.41 | 0.79 | 0.33 |

BSID-III: Bayley Scales of Infant Development III

IFA: Iron-folic acid supplements

LNS: Lipid-based nutrient supplements

MMS: Multiple micronutrient supplements

**Table I.** Multivariable* analyses of the effect of MMS and LNS on time to achievement of the WHO gross motor milestones as compared to IFA.

|  | **MMS vs. IFA Hazard ratio for achievement* (95% CI)** | **p-value** | **LNS vs. IFA Hazard ratio for achievement* (95% CI)** | **p-value** |
| --- | --- | --- | --- | --- |
| Sitting without support | 1.18 (0.73, 1.92) | 0.49 | 1.37 (0.89, 2.11) | 0.15 |
| Standing with assistance | 1.07 (0.67, 1.71) | 0.79 | 1.35 (0.89, 2.06) | 0.16 |
| Hands-and-knees crawling | 1.13 (0.69, 1.86) | 0.62 | 1.36 (0.89, 2.08) | 0.16 |
| Walking with assistance | 1.01 (0.66, 1.54) | 0.97 | 1.38 (0.96, 2.01) | 0.09 |
| Standing alone | 1.11 (0.75, 1.63) | 0.61 | 1.53 (1.12, 2.09) | 0.01 |
| Walking alone | 1.07 (0.76, 1.49) | 0.71 | 1.49 (1.15, 1.93) | <0.001 |

*Multivariable adjusted estimates control for household wealth quintile, household size, food security, maternal age (in years), education (any vs. none), anemia (Hb<11 g/dL vs. Hb ≥11 g/dL), underweight (BMI <18.5 kg/m2 vs. BMI ≥18.5 kg/m2), malaria (positive RDT vs. negative RDT), child sex, and season of enrollment in the trial (hunger vs. not hunger season). Missing indicators were used to retain participants. Models accounted for clustering at the village level.

BSID-III: Bayley Scales of Infant Development III

IFA: Iron-folic acid supplements

LNS: Lipid-based nutrient supplements

MMS: Multiple micronutrient

**Table J.** Modeled means and standard errors for BSID-III cognitive scores at 6, 9, 12, 15, 18, 21, and 24 months by for LNS versus IFA (p-value for difference in test of trajectory <0.001)

|  | IFA modeled mean ± SE BSID-III cognitive score | LNS modeled Mean ± SE BSID-III cognitive score | p-value* for difference in LNS compared to IFA at time point |
| --- | --- | --- | --- |
| Month 6 | 31.4 ± 0.4 | 30.8 ± 0.4 | 0.33 |
| Month 9 | 41.0 ± 0.8 | 40.0 ± 0.2 | 0.25 |
| Month 12 | 47.1 ± 1.1 | 46.9 ± 0.3 | 0.87 |
| Month 15 | 51.4 ± 0.5 | 51.9 ± 0.2 | 0.38 |
| Month 18 | 55.1 ± 0.2 | 56.5 ± 0.4 | 0.001* |
| Month 21 | 57.5 ± 0.5 | 58.9 ± 0.4 | 0.02* |
| Month 24 | 59.1 ± 0.9 | 60.0 ± 0.3 | 0.25 |

*Statistically significant with Tukey-Kramer method for multiple comparisons alpha =0.026

IFA: Iron-folic acid supplements

LNS: Lipid-based nutrient supplements

**Fig A.** Survival plot for time to achievement of sitting without support stratified by randomized group


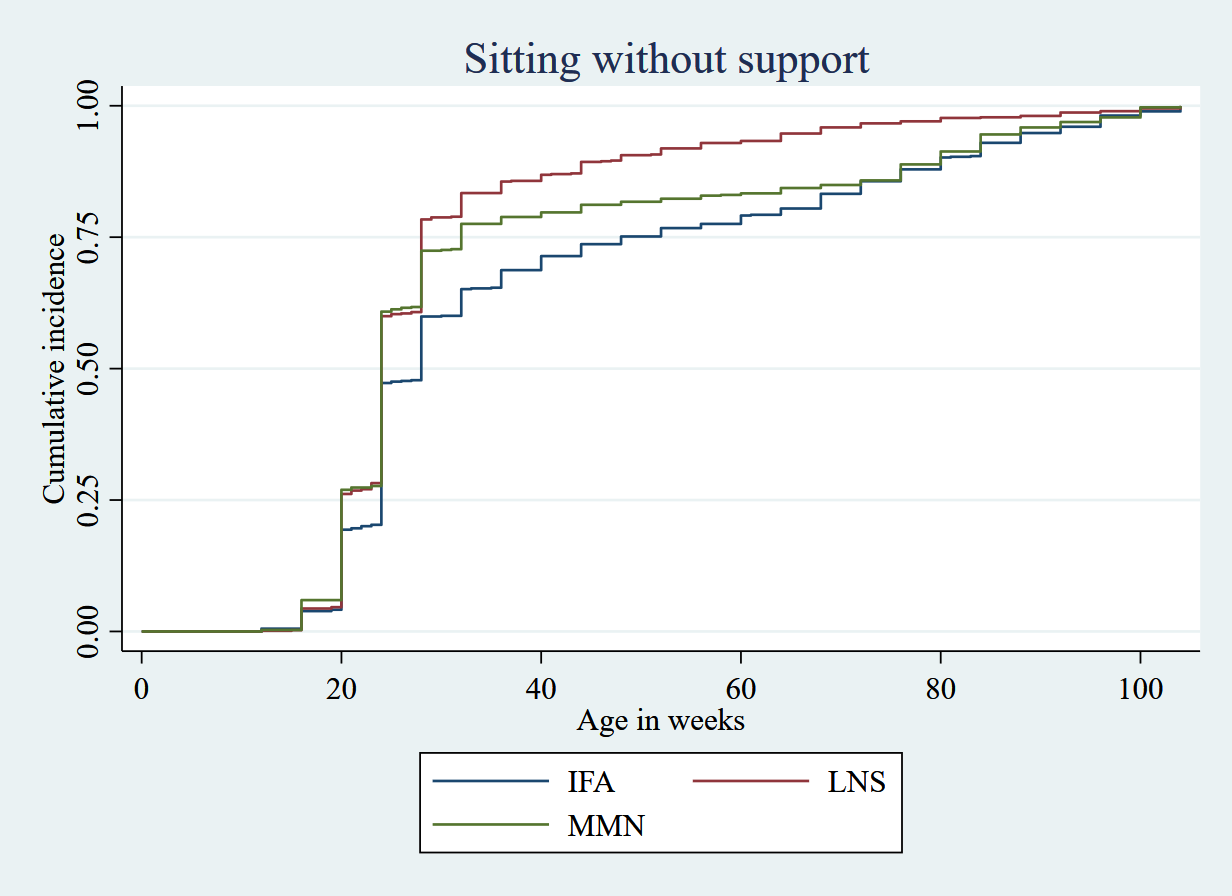


IFA: Iron-folic acid supplements

LNS: Lipid-based nutrient supplements

MMN: Multiple micronutrient supplements

**Fig B.** Survival plot for time to achievement of standing with assistance stratified by randomized group


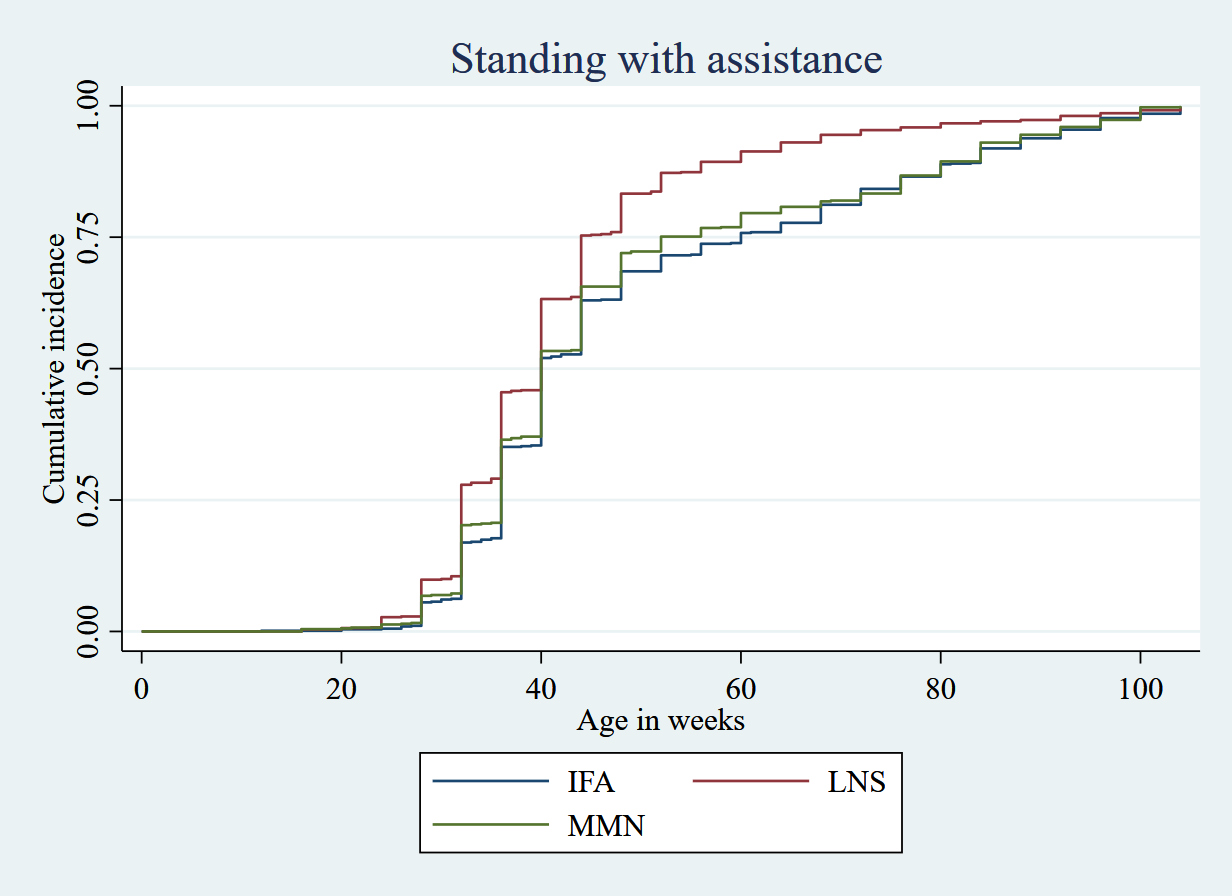


IFA: Iron-folic acid supplements

LNS: Lipid-based nutrient supplements

MMN: Multiple micronutrient supplements

**Fig C.** Survival plot for time to achievement of hands-and-knees crawling stratified by randomized group


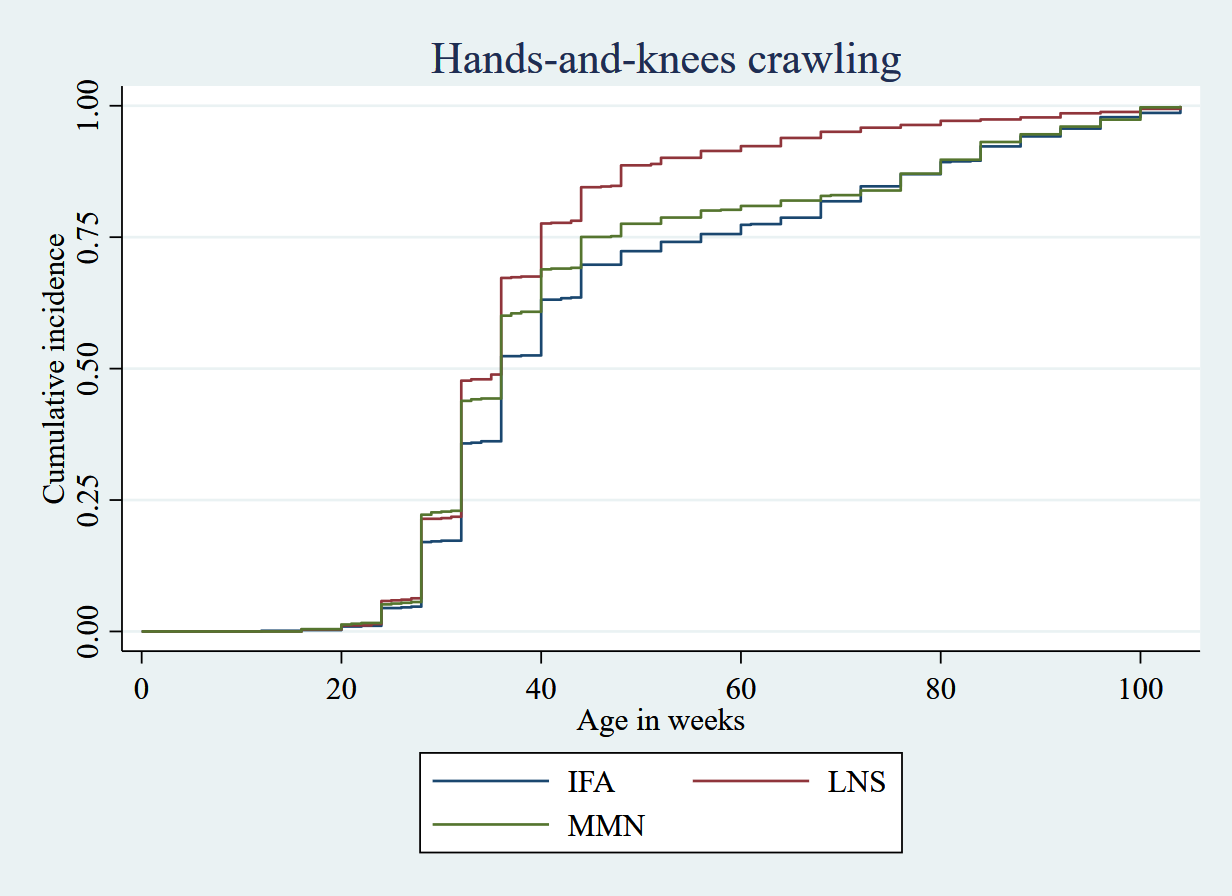


IFA: Iron-folic acid supplements

LNS: Lipid-based nutrient supplements

MMN: Multiple micronutrient supplements

**Fig D.** Survival plot for time to achievement of walking with assistance stratified by randomized group


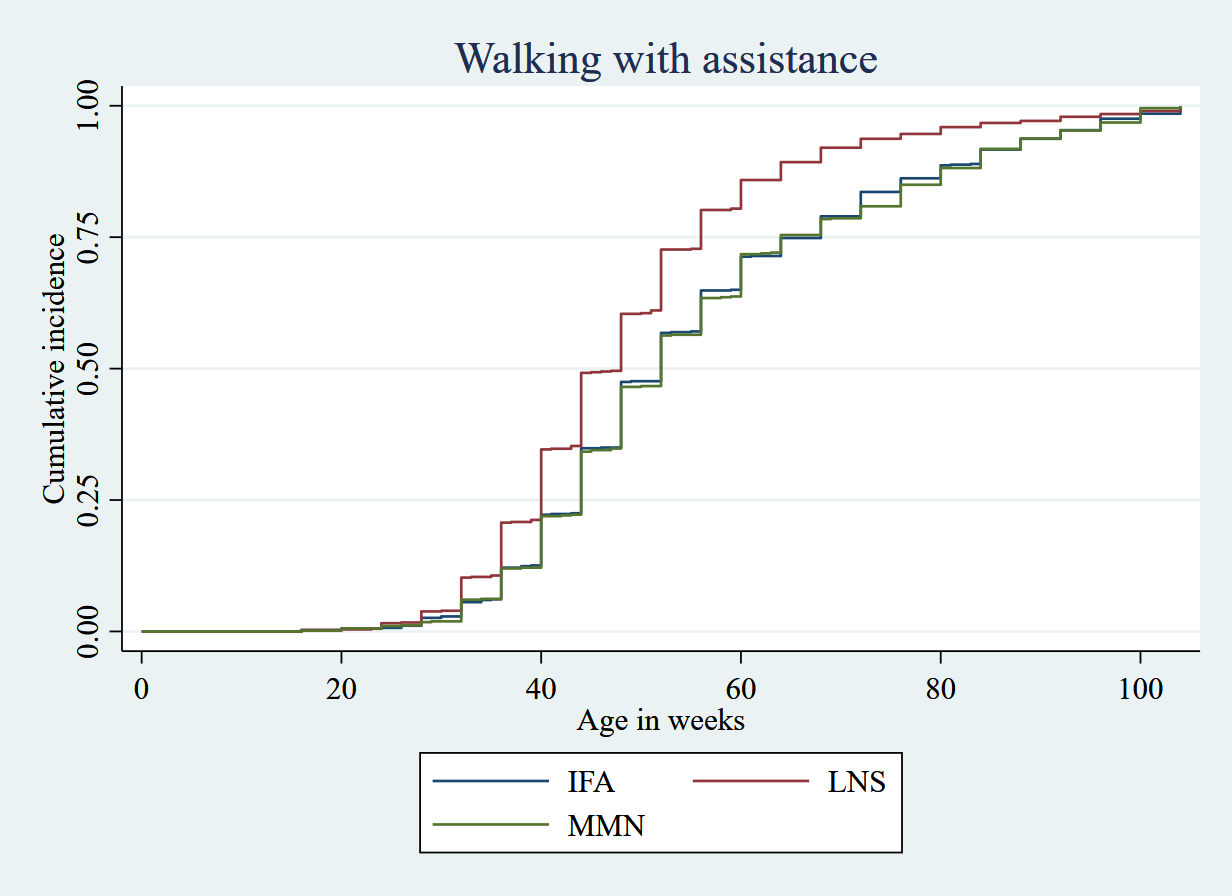


IFA: Iron-folic acid supplements

LNS: Lipid-based nutrient supplements

MMN: Multiple micronutrient supplements

**Fig E.** Survival plot for time to achievement of standing alone stratified by randomized group


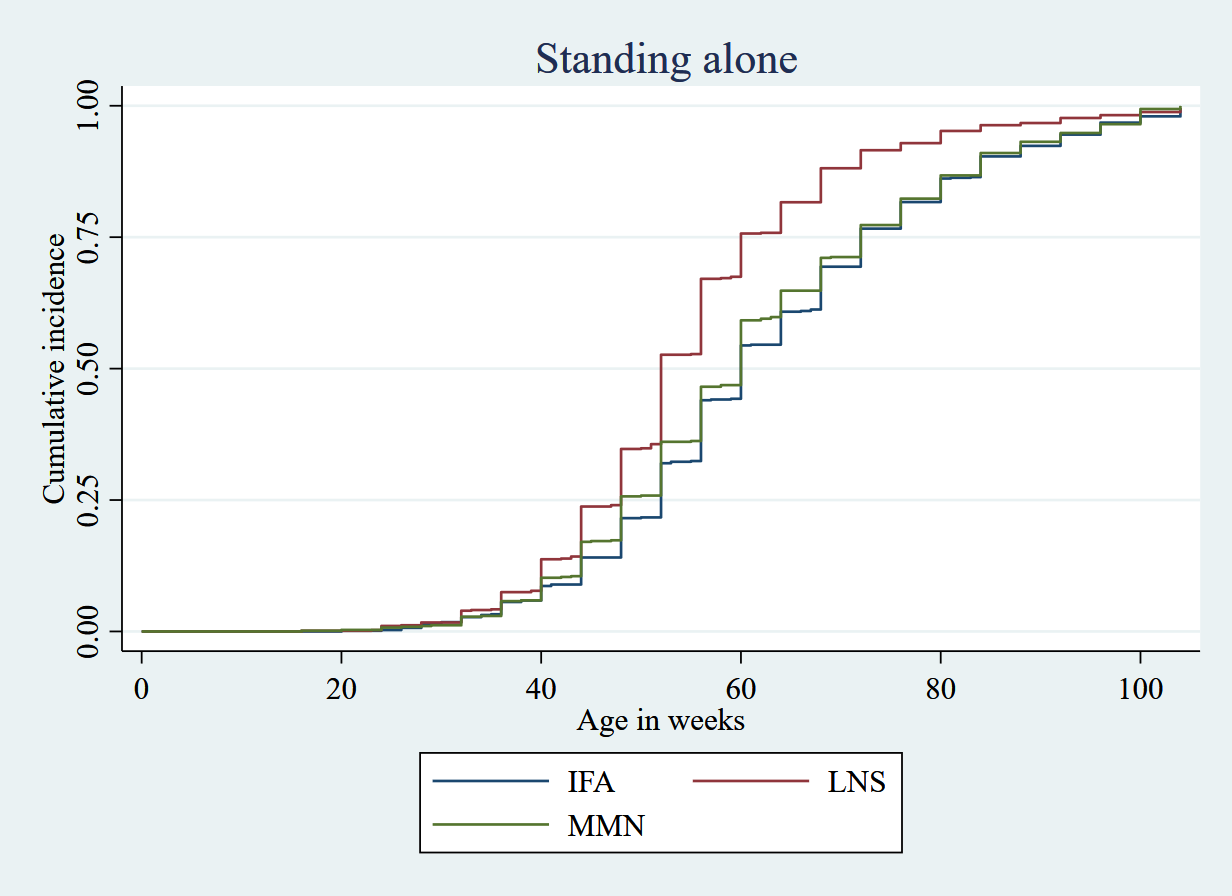


IFA: Iron-folic acid supplements

LNS: Lipid-based nutrient supplements

MMN: Multiple micronutrient supplements

**Fig F.** Survival plot for time to achievement of walking alone stratified by randomized group


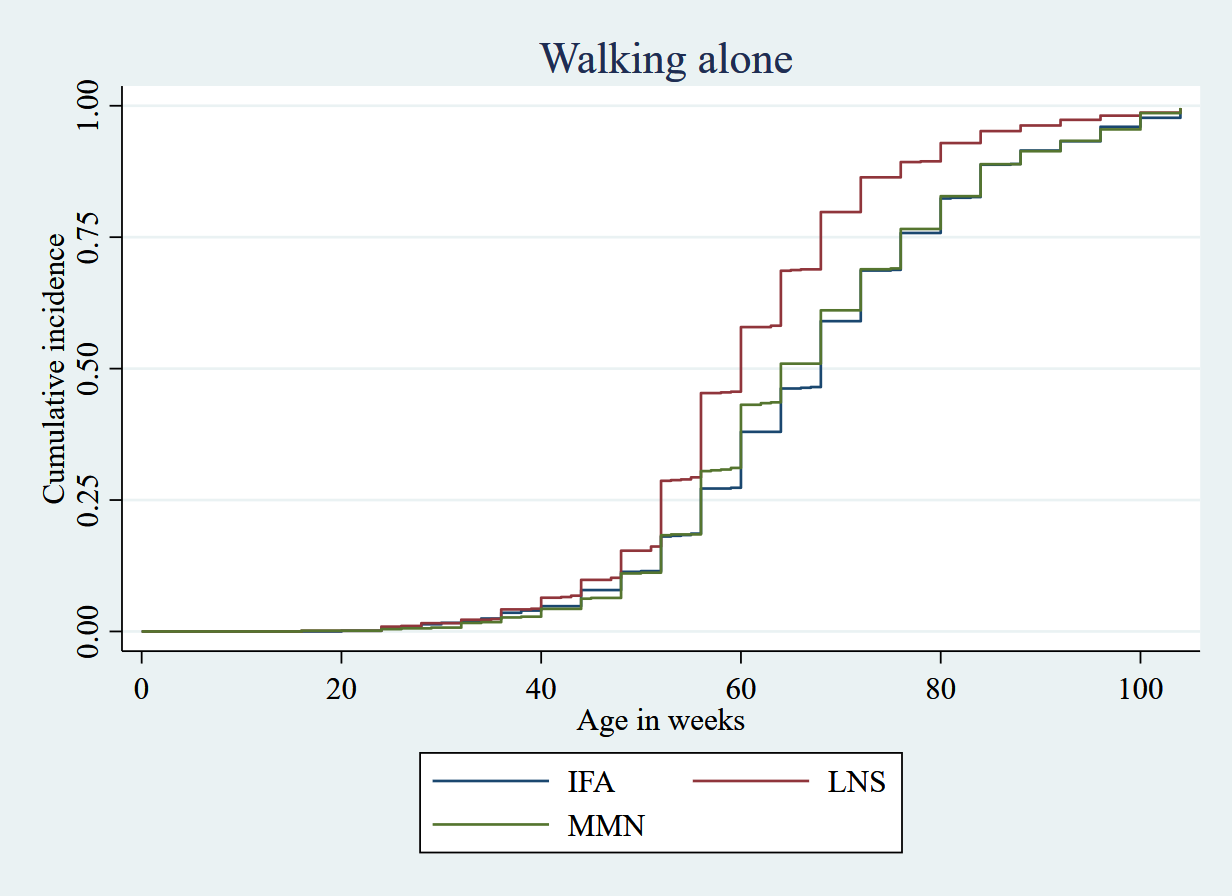


IFA: Iron-folic acid supplements

LNS: Lipid-based nutrient supplements

MMN: Multiple micronutrient supplements
